# A new method to measure EC_50_ reveals cultivar‐specific fungicide resistance and very high diversity within experimental field populations of *Zymoseptoria tritici*

**DOI:** 10.1002/ps.70483

**Published:** 2026-01-19

**Authors:** Firas Talas, Jessica Stapley, Bruce A McDonald

**Affiliations:** ^1^ Plant Pathology Group, Institute of Integrative Biology ETH Zurich Zürich Switzerland

**Keywords:** EC_50_, host–fungicide–pathogen interactions, resazurin, Septoria tritici blotch

## Abstract

**BACKGROUND:**

*Zymoseptoria tritici* causes Septoria tritici blotch (STB), the most damaging wheat disease in Europe. In Europe, STB is controlled mainly by fungicides and fungicide resistance is frequently reported. Although fungicide resistance is thought to emerge mainly from standing genetic variation within field populations of *Z. tritici*, few studies have attempted to quantify the degree of fungicide resistance occurring at the field scale and to measure changes in the frequencies of resistant strains following fungicide applications during a single growing season. Even fewer studies have considered the effects of different wheat cultivars on the emergence of fungicide resistance. We developed a new high‐throughput method based on resazurin dye and image analysis to measure the effective concentration of a fungicide that reduces growth by 50% (EC_50_) values and applied it to 1005 strains of *Z. tritici* sampled at two time points from 17 different wheat cultivars growing in a replicated field experiment. The experimental field was treated with combinations of five different active ingredients at three times during the growing season.

**RESULTS:**

We found that field populations of *Z. tritici* can maintain a very high diversity in fungicide sensitivity phenotypes despite three fungicide treatments, with as much diversity found within a single field during a single growing season as has been described across all of Europe over several years. We discovered that wheat cultivars that were more resistant to STB tended to be colonized by *Z. tritici* strains that exhibited higher fungicide resistance. We also found that specific wheat cultivars selected for resistance to specific active ingredients, providing the first direct support for the existence of significant host–fungicide–pathogen interactions.

**CONCLUSION:**

Overall, our findings illustrate the many challenges associated with designing fungicide treatment programs that aim to reduce selection for fungicide resistance when confronted with a pathogen like *Z. tritici* that has a very high evolutionary potential. © 2026 The Author(s). *Pest Management Science* published by John Wiley & Sons Ltd on behalf of Society of Chemical Industry.

## Graphical abstract

We isolated *Zymoseptoria tritici* strains from a diverse wheat field and quantified fungicide resistance using a novel plate assay. We found high diversity in fungicide sensitivity and host–fungicide–pathogen interactions.

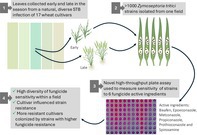

## 1 INTRODUCTION

*Zymoseptoria tritici*, the pathogen causing Septoria tritici blotch (STB), poses a substantial threat as a destructive wheat pathogen, especially in Europe where it typically causes yield losses of 5–10% despite the intensive use of fungicides and deployment of resistant cultivars.^1^ Approximately 70% of all fungicide applications on wheat in Europe are aimed toward controlling STB.^2^ *Z. tritici* populations have evolved resistance to fungicides in all regions where they are regularly applied, including the triazoles metconazole, epoxiconazole, and propiconazole,^3^ and the succinate dehydrogenase inhibitor (SDHI) bixafen.^4^ The rapid emergence of fungicide resistance is facilitated by the high evolutionary potential of *Z. tritici*, which reflects its frequent sexual recombination, high effective population size, and substantial gene flow across regional scales.^5^ A recent analysis^6^ indicated that each hectare of a wheat field with a moderate degree of STB infection (~40% of leaves infected) contains ~8 million different pathogen genotypes that produce ~5 trillion pycnidiospores during a typical growing season, with ~70 million of these spores expected to encode mutations for fungicide resistance. These findings indicate that most fungicide resistance is likely to emerge from standing genetic variation in fields affected by STB and that the rate of resistance evolution is unlikely to be mutation‐limited. This suggests that integrated strategies are needed to slow the emergence of fungicide resistance.

One common integrated strategy is simultaneous deployment of resistant cultivars and fungicides within the same field. If host resistance and fungicides act independently against the pathogen, they can theoretically offer mutual protection against the evolution of fungicide resistance as well as the breakdown of host resistance (e.g., see modeling studies by Taylor and Cunniffe^7^ and Carolan *et al*.^8^). But if host resistance and fungicides are not independent, e.g. if there are significant interactions among host cultivars, fungicide active ingredients, and pathogen strains (referred to here as H–F–P interactions), then the hypothesized advantages may not be realized. Experimental investigation of H–F–P interactions remains rare because it requires precise measurements of fungicide sensitivity for a large number of pathogen strains across a broad array of host genotypes. Such investigations are costly and remain impractical until precise and high‐throughput methods for measuring the relevant phenotypes become available. Here, we describe development of a high‐throughput method for measuring the effective concentration of a fungicide that reduces growth by 50% (EC_50_) in *Z. tritici* which complements our earlier development of high‐throughput methods for measuring quantitative resistance to STB^9^, ^10^ and quantitative virulence in *Z. tritici*.^10^, ^11^

Several approaches have been developed to evaluate fungicide sensitivity *in vitro*, including calculation of minimum inhibitory concentration (MIC),^3^ differences in growth rates among isolates,^12^ and the disk diffusion method.^13^ EC_50_ calculated across several fungicide concentrations has emerged as the most comprehensive measure of fungicide efficacy because it allows for more robust comparisons between different studies. For example, Marciano and Toffolatti^14^ compared EC_50_ values obtained using Petri dishes, 24‐well, and 96‐well plates for *Phytophthora infestans* and reported highly similar outcomes, supporting the idea that maintaining a consistent range of doses and using the same statistical method can provide highly correlated results. McNab *et al*.^15^ tested the sensitivity of 30 isolates of *Clarireedia jacksonii* to propiconazole and found that the transformed EC_50_ values obtained using microtiter plates were highly correlated (*R* ^2^ = 0.56, *P* < 0.001) with values obtained using an amended agar assay. However, EC_50_ measurements require more extensive data than other measures, and EC_50_ calculations are more complex because they follow a nonlinear model. Several studies have already used EC_50_ to measure fungicide efficacy in *Z. tritici*,^16^, ^17^, ^18^ and other fungi by measuring growth rates based on changes in optical density (OD) in microtiter plates containing serial dilutions of fungicides.^16^, ^19^, ^20^ This approach works best for fungi that exhibit yeast‐like growth. However, a pathogen such as *Z. tritici* tends to produce variable amounts of melanin and can switch from blastospore growth to mycelial growth under stressful conditions,^21^ which can affect the accuracy of OD measurements. For pathogens like *Z. tritici*, a non‐toxic redox dye called resazurin (RZ) can provide an alternative measure of growth in microtiter plates^22^, ^23^ that is unaffected by changes in growth morphology or melanin production. RZ has a blue color that irreversibly turns pink upon exposure to metabolic activity and currently provides a routine bioassay for measuring cytotoxicity.^24^ A limitation of RZ is that it is sensitive to low pH levels (pH 6.5–4.5), where the blue color can instead change to colors that range from orange to transparent, respectively.^25^ RZ in microtiter plates has already been used to measure fungicide efficacy for *Aspergillus* spp. infecting humans,^26^ for *Alternaria alternata* infecting crops,^23^, ^27^ and for *Monilinia fructicola* on fruit trees.^28^ Here we describe a method using RZ to measure EC_50_ in *Z. tritici*.

Although we are not aware of other studies specifically addressing the possibility of H–F–P interactions in plant pathosystems, several studies in *Z. tritici* have found evidence that host genotype can affect the degree of fungicide sensitivity found in pathogen strains infecting that host. The first evidence for a significant H–F–P interaction was reported in Yang *et al*.,^29^ where a significant difference was found in resistance to cyproconazole among *Z. tritici* populations sampled from a resistant and a susceptible wheat cultivar growing in the same untreated field. More evidence for significant H–F–P interactions came from extensive phenotyping of 145 global strains of *Z. tritici* that revealed significant negative genetic correlations between propiconazole resistance and both virulence and pathogen reproduction for 3 of the 12 tested wheat cultivars.^10^ But until now, we are not aware of any experiments specifically designed to measure and detect potential H–F–P interactions.

Here, we describe an experiment suitable for directly detecting H–F–P interactions under field conditions and report strong evidence for significant H–F–P interactions in STB. We conducted an extensive investigation of fungicide resistance in a unique collection of *Z. tritici* isolates assembled from an experimental field in Eschikon, Switzerland. The isolates were sampled in 2016 from a single field in which 335 elite European winter wheat cultivars were naturally infected by STB despite intensive applications of fungicide mixtures containing five different active ingredients.^10^ By collecting strains at different points in time from known hosts growing in the same field, we could investigate several processes associated with fungicide resistance. In particular, the current work aimed to:

1. Quantify the diversity of fungicide resistance phenotypes that exist within *Z. tritici* populations in fungicide‐treated fields and determine whether this diversity changes over the course of a growing season.
2. Determine whether host genotype affects resistance to different active ingredients, i.e. is there evidence for significant H–F–P interactions?

To meet these aims, we developed a new high‐throughput method using RZ dye, flatbed scanners, and image analysis to measure EC_50_ for six fungicides in more than 1000 strains of *Z. tritici* obtained from 17 of the wheat cultivars.

## 2 MATERIALS AND METHODS

### 2.1 Fungal material and field experiment

A replicated field experiment was conducted to evaluate quantitative resistance to STB in 335 elite winter wheat (*Triticum aestivum*) varieties grown under field conditions.^9^ The experiment was conducted in two biological replicates during the 2015–2016 growing season, with the two replicates separated by ~100 m in the Eschikon field station of ETH Zurich, Switzerland (coordinates 47.449° N, 8.682° E). All wheat cultivars were infected naturally by the local population of *Z. tritici*, with many primary infections likely coming from airborne ascospores originating from other wheat fields growing in the area. All 1.2 × 1.7 m plots were sown on 13 October 2015. The plots were treated three times with different fungicidal mixtures, namely (i) on 6 April 2016: Input®, Bayer (a mix of spiroxamine at 300 g L^−1^ and prothioconazole at 150 g L^−1^, at a dose of 1.25 L ha^−1^, at growth stage (GS) 31^30^; (ii) on 25 May 2016: Aviator® Xpro, Bayer (a mix of bixafen at 75 g L^−1^ and prothioconazole at 150 g L^−1^, at a dose of 1.25 L ha^−1^, GS51); and (iii) on 6 June 2016: Osiris®, BASF (a mix of epoxiconazole at 56.25 g L^−1^ and metconazole at 41.25 g L^−1^, at a dose of 2.5 L ha^−1^, GS65 that corresponds to anthesis). Sixteen leaves showing symptoms of STB were sampled randomly from each plot at two times during the season (32 leaves in total). The first collection (C1) was sampled on 20 May at GS41 and a third collection (C3) was sampled on 4 July at GS75–85. A second smaller collection (C2) sampled on 17 June was not included in these analyses. At C1, infected leaves were collected from the highest infected leaf layer, which was typically the third or fourth fully extended, but non‐senescent leaf still visible when counting from the ground, or one to three leaf layers below the flag leaf. At C3, the leaf layer below the flag leaf was sampled in each plot. Groups of eight leaves were mounted on A4 paper, scanned, and analyzed using ImageJ^31^ to measure the percentage of leaf area covered by lesions (PLACL), pycnidia density within lesions (ρlesions), and pycnidia density within leaves (ρleaf^9^) as quantitative indicators of resistance to STB. After ranking all 335 cultivars for their quantitative resistance to STB, a sample of 17 cultivars reflecting the entire range of STB resistance were selected to make pathogen isolations.

To isolate *Z. tritici* from these leaves, a cirrus from a single pycnidium was plated on a Petri dish containing yeast malt sucrose agar with kanamycin (50 μg.mL^−1^). After incubating at 18 °C for 7 days, a single‐spore colony was transferred to 35 mL of yeast sucrose broth (YSB, 10 g of sucrose and 10 g of yeast extract in 1 L of ddH_2_O + 50 mg L^−1^ streptomycin +50 mg L^−1^ chloramphenicol) and placed on an orbital shaker (180 rpm) for 7 days at 18 °C. A dense concentration of blastospores was preserved on silica gel and stored at −80 °C for future use.^32^ To recover the isolates from long‐term storage, dried spores were transferred into 20 mL of YSB. After 7 days of incubation at 180 rpm at 18 °C in the dark, the blastospore suspension was filtered through two layers of sterile cheesecloth, then centrifuged at 3500 rpm (2851*g*) at 4 °C for 15 min. The spore pellet was resuspended with sterile dd H_2_O and the blastospore concentration was adjusted to 8 × 10^5^ spore mL^−1^ using KOVA (KOVA International, Garden Grove, CA, USA) counting slides and a light microscope. In total, 410 of the isolates from C1 and 595 of the isolates from C3 were used in the experiments of fungicide efficacy reported here.

### 2.2 Microtiter plate assays

We tested fungicidal effects in sterile 96‐well microtiter plates (Thermo Fisher Scientific, Nunc^TM^, Waltham, MA, USA). Each *Z. tritici* isolate was added at the same concentration (8 × 10^5^ blastospores.mL^−1^) to a column of eight wells; seven with YSB media containing a fungicide diluted to seven concentrations and one control well containing YSB media but no fungicide. Diluted RZ dye was added into the YSB–fungicide mixture before adding blastospores. Every isolate was replicated three times in different microtiter plates. Each plate included one control column that did not receive any blastospores (with no fungal growth). The position of the control column was completely randomized with respect to the other 11 isolate‐containing columns on the same plate. Plates were sealed with Parafilm, labeled with a unique QR code, and incubated at 18 °C for 7 days in the dark and at 60% relative humidity to minimize the evaporation of liquid from the plates. The plates were distributed on metal mesh shelves in an incubator (Kälte 3000 AG, 2008, Switzerland) using a completely randomized design. After 7 days, we scanned the plates and the associated QR code using a flatbed scanner at 600 dpi.

### 2.3 Calibration of RZ

RZ dye (Sigma‐Aldrich, Merck KGaA, St. Louis, MO, USA) was dissolved in sterile ddH_2_O and sterilized through a 45‐μm filter to a final concentration of 5000 mg.l^−1^. Two *Z. tritici* strains chosen at random from different cultivars in the first collection, c1_25_2B2 and c1_290_9H1, were used to conduct this calibration experiment. *Z. tritici* metabolizes RZ to resorufin, causing a color change from blue to red. To determine the optimum concentrations of RZ and blastospores to use in the main experiments, we implemented a two‐dimensional design: serial dilutions of spores were applied across the rows, and serial dilutions of RZ were applied across the columns. The tested concentrations of RZ were 350, 300, 250, 200, 180, 150, 100, 80, 50, 40, 30, and 20 mg L^−1^, and the tested concentrations of blastospores were 1 × 10^5^, 2 × 10^5^, 4 × 10^5^, 6 × 10^5^, 8 × 10^5^, 10 × 10^5^, 12 × 10^5^ and 15 × 10^5^ spores mL^−1^, providing 96 different combinations of RZ and blastospores in each plate. This optimization experiment was replicated twice for each isolate. Our goal was to determine the lowest concentration of RZ that would detect the greatest range in color change (to maximize the ability to distinguish between different EC_50_ values) as a measure of fungal metabolism that could be used as a proxy of fungal growth. The plates were scanned using a flatbed scanner at different times (1, 24, 48, 72, 96, 120, 144, and 168 h after adding spores) to measure the amount of red color produced in response to fungal metabolism. The optimization of experimental conditions was analyzed using SAS software.^33^ The exponential (log) growth phase represents the most homogeneous and experimentally reproducible phase of fungal development, during which the population tends to exhibit a normal distribution in growth characteristics. By combining gradients of both spore and RZ concentrations, we were able to detect this normal distribution both visually and statistically. This normal distribution serves as a proxy for identifying the optimal incubation time, while simultaneously allowing us to determine the minimum RZ concentration required to visualize fungal growth. This approach helps to ensure that any metabolic effect of RZ is minimized, although previous studies showed that such effects are negligible at low concentrations.^34^

### 2.4 Measuring fungicide efficacy

We used the RZ as a metabolic indicator of the amount of *Z. tritici* growth in each well based on measuring the induced red color using digital image analysis. The serial dilution of each fungicide was adjusted to fill one column in the microtiter plate with eight different concentrations. We tested six active ingredients (AIs) in total: the five AIs applied to the field experiment in which the *Z. tritici* isolates originated (prothioconazole, metconazole, epoxiconazole, spiroxamine, and bixafen), and propiconazole, a triazole fungicide that was first applied to European wheat fields approximately 40 years ago. The tested fungicide ranges were chosen to enable some growth of fungal isolates at low concentrations while aiming to achieve no growth or minimal growth at the highest concentration. We used the following serial dilutions: 0, 3.75, 7.5, 18.75, 37.5, 112.5, 150, and 225 mg L^−1^ for propiconazole and prothioconazole; 0, 3.75, 7.5, 18.75, 37.5, 75, 112.5, and 150 mg L^−1^ for metconazole; 0, 0.75, 3.75, 7.5, 18.75, 37.5, 75, and 187.5 mg L^−1^ for epoxiconazole; 0, 0.08, 0.38, 0.75, 1.5, 3.75, 7.5, and 75 mg L^−1^ for spiroxamine; and 0, 0.008, 0.075, 0.375, 0.75, 1.5, 7.5, and 75 mg L^−1^ for bixafen.

After analyzing the complete data set for all isolates, we identified subsets of 52, 53, 88, 70, 29, and 3 isolates for propiconazole, prothioconazole, metconazole, epoxiconazole, spiroxamine, and bixafen, respectively that required either a higher or lower range of fungicide concentrations to establish their EC_50_ values. We retested those isolates using a different range of serial dilutions as described in Supporting Information, Table S1.

### 2.5 Analyzing the digital images

We created a series of shell and ImageJ Macro (IJM) scripts to create and read the QR codes, sort the images according to the plate name (the active ingredient, replicate, and date of testing), and analyze the red color intensity in each well of each microtiter plate (Supporting Information, [Link], [Link] and S3). The red color intensity was used as a proxy for the fungal biomass in each well. We modified an existing macro for reading red color intensity in microtiter plates^35^ (Supporting Information, File S3). Each well in a plate was identified and labeled based on its position in the plate. Then each well image was analyzed to separate its color components (red, green, and blue) and the red intensity of each pixel was calculated on a scale of 0–255 using ImageJ.^35^, ^36^ The degree of redness mirrors the intensity of the metabolic activity of the fungus (the higher its metabolism, the more intense the red color) and the metabolic activity was used as a proxy of the total fungal biomass present in each well. Based on the well position in the plate, we could analyze red intensity values as a function of fungicide concentration.

### 2.6 Comparison of biomass values calculated using RZ and OD measurements

We chose 22 of the strains to make a direct comparison between the biomass values calculated using our new RZ method with conventional OD measurements. OD measurements were obtained at a wavelength of 570 nm in a Tecan Infinite plate reader for strains growing in two microtiter plates containing different sets of fungicide concentrations after 168 h of growth. The correlations between the biomass values calculated using RZ and OD were estimated using a simple linear regression model, providing correlation coefficients and *P* values.

### 2.7 Calculation and analysis of EC_50_ values

The EC_50_ value was calculated based on the growth of each isolate over eight concentrations by applying a nonlinear model with four parameters using the following formula:

y=min+max−min/1+10^logEC50−x×Hillslope

Where y= the isolate response to the active ingredient (measured by the redness value as an indicator of fungal growth), min = minimum red value within the active ingredient spectrum of the isolate under investigation (at the highest AI concentration), max= maximum red value within the active ingredient spectrum of the isolate under investigation (at the zero AI concentration), EC_50_ is the concentration of active ingredient that can suppress the metabolic activity (growth) of a fungal isolate by 50%, and x= the concentration of active ingredient corresponding to y fungal growth. The individual red values of each replicate were integrated into the model, hence the average value of each data point (i.e. AI concentration) was used to generate the EC_50_ curve and the fitted EC_50_ value. SPSS software^37^ was used to measure the correlation in EC_50_ across replicates. The remainder of the analyses and plotting of results was performed with R software^38^ and R Studio.^39^ The EC_50_ values were normalized (*z*_𝑖_ = 𝑥_𝑖_ − min(𝑥)/(max(𝑥) − min(𝑥)) because the scales varied for each AI (e.g. for bixafen values were < 10, whereas for propiconazole values ranged up to 161) and log‐transformed to meet the assumptions of linear models. To investigate the effect of collection time, host cultivar and AI on EC_50_ values two approaches were used: the first used the value for each technical replicate as the response variable and included replicate in the model as an explanatory variable (y ~ collection + replicate + host); the second used the mean value across the three technical replicates as the response variable (y ~ collection + host). Although the first approach is less conservative, it takes into account possible microenvironment effects during incubation and may reveal more subtle effects because of the three times larger number of observations. After fitting linear models and performing an analysis of variance, we performed multiple pairwise comparisons using least significant differences (LSD) and adjusted the *P* values for multiple testing using the Bonferroni correction. To test for the effect of collection time, host cultivar and the interaction between AI and host cultivar, the mean EC_50_ across three replicates was used as the response variable, while collection time, host, AI and the interaction between host and AI were explanatory variables.

### 2.8 Broad‐sense heritability

To estimate broad‐sense heritability (*H* ^2^ a measure of how much of the variance in EC_50_ values is due to genetic differences among strains), we used a linear mixed effect (LME) model and variance partitioning. For the LME, the log‐transformed and normalized EC_50_ value was the response variable, collection time was the fitted factor, and random factors were host cultivar, AI, replicate and isolate. Variance components were extracted and used to calculate *H* ^2^.

### 2.9 Correlation between cultivar resistance to STB and fungicide sensitivity

The correlation between the EC_50_ values for each AI and the degree of resistance to STB on each wheat cultivar was evaluated using three quantitative resistance measures: the percentage of leaf area covered by lesions (PLACL), the density of pycnidia per cm^2^ of lesion area (ρlesions), and the density of pycnidia per cm^2^ of leaf area (ρleaf). Pearson correlations were calculated in R software^38^ using R Studio.^39^

## 3 RESULTS

### 3.1 Developing a digital image analysis pipeline

The microtiter plates with RZ showed different gradients of colors which reflected the cumulative growth of each isolate across the serial dilutions of each active ingredient. The uninoculated control column in each plate remained blue and did not show any change in color across all eight concentrations over the entire experiment.

**Figure 1:**
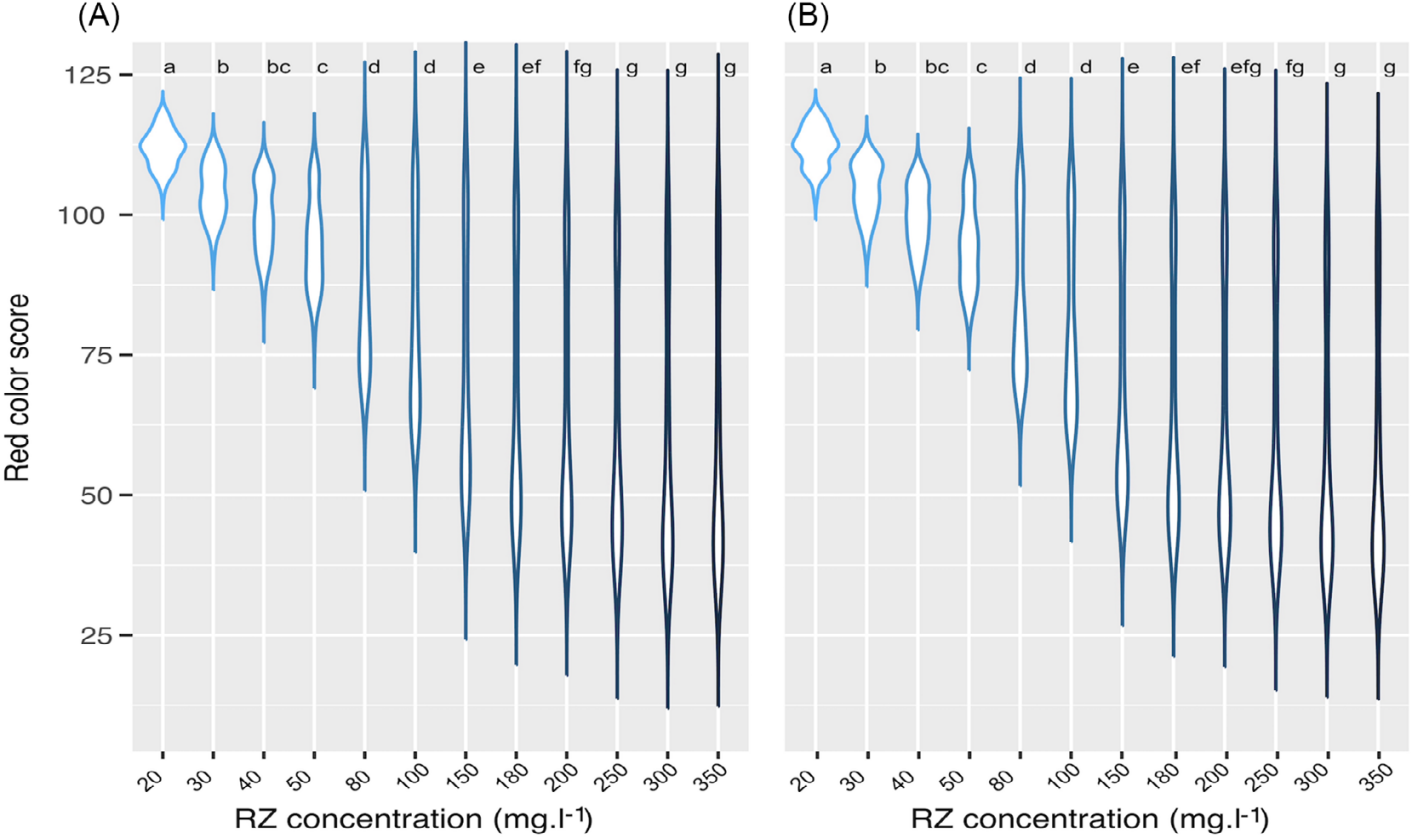
Optimizing resazurin (RZ) concentration through a factorial experiment using two isolates of *Zymoseptoria tritici*; c1_25_2B2 (A) and c1_290_9H1 (B), each with two repetitions. After applying a Bonferroni correction, the least significant difference (LSD) was used to determine differences among treatments, indicated with letters at the top of each tested RZ concentration. The concentration range of 180–200 mg L^−1^ was identified as the minimum level of RZ that captured a significant proportion of the red color spectrum, indicative of fungal metabolic activity.

**Figure 2:**
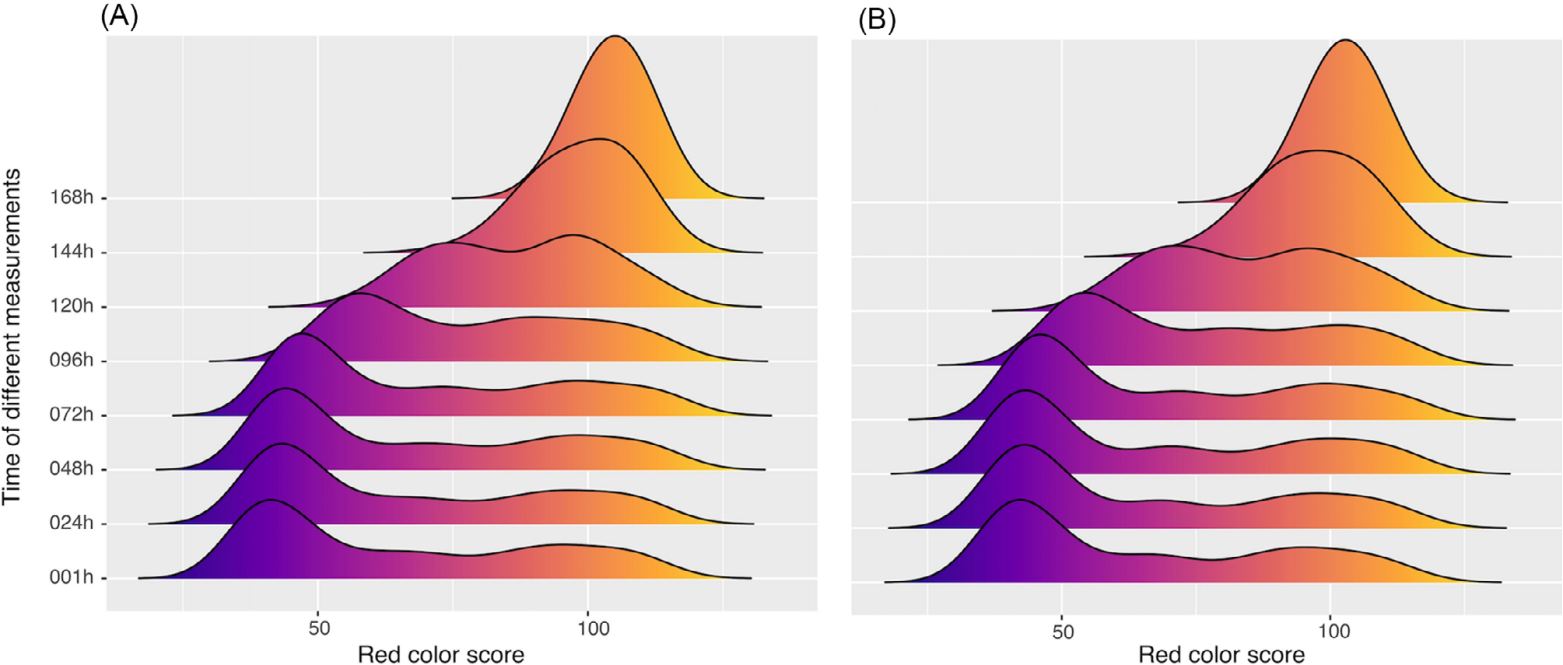
Optimizing the incubation time for data collection in a factorial experiment involving two strains of *Zymoseptoria tritici*; c1_25_2B2 (A) and c1_290_9H1 (B), each with two repetitions. The experiment, conducted in 96‐well microtiter plates, included a horizontal gradient composed of serial dilutions of resazurin and a vertical gradient composed of serial dilutions of blastospores. Red color intensity was measured at eight different time points for each microtiter plate. Each time point includes the complete distribution of red color scores across all 96 combinations of resazurin and blastospore concentrations. The mean values of both replicates were plotted on the *x*‐axis, revealing quantitative, gradual changes from blue to red. A normal distribution was observed at the optimal incubation time of 168 h.

We tested 12 different concentrations of RZ in the absence of fungicides and measured the red color scores across 1 to 168 h of incubation. We found significant variation among the RZ concentrations for both tested isolates (Fig. 1). A concentration between 180 and 200 mg L^−1^ was chosen as the lowest RZ concentration that covered a significant fraction of the red color spectrum. Color densities (gradual changes from blue to red relative to varying concentrations of RZ and blastospores) also varied over time. More than one peak can be seen between the 1 and 120 h post‐inoculation periods (Fig. 2). Although we found a single peak at 144 h, a more normal distribution was exhibited by the peak seen at 168 h post‐inoculation (Fig. 2). Based on these findings, we chose 168 h after inoculation as an optimal time point for collecting the data. Analysis of variance revealed significant variation among blastospore concentrations and RZ concentrations, but no significant differences were found between replications (Table 1). An LSD test of the blastospore concentrations indicated three significantly different categories, leading us to choose the intermediate group (8 × 10^5^–12 × 10^5^ spores mL^−1^) (Supporting Information, Fig. S1) for the main experiment. In summary, these findings led us to choose 168 h of incubation, 180 mL L^−1^ of RZ, and 8 × 10^5^ blastospores per mL as the optimum conditions to measure fungicide sensitivity.

**Table 1:** Analysis of variance for the effects of resazurin (RZ) concentration and spore concentration on red color intensity in a microtiter plate assay of fungal metabolic activity

Source	df	Sum of Squares	Mean of Squares	*F* value	Pr(>*F*)	
Isolate	1	1239	1239	4.55	0.033	*
Spore concentration	7	25 208	3601	13.226	<2 × 10^−16^	***
RZ concentration	11	1 064 252	96 750	355.341	<2 × 10^−16^	***
Replication	1	1	1	0.004	0.948	
Residuals	3051	830 709	272			

*Note*: *P < 0.05, **P < 0.01, ***P < 0.001.

### 3.2 Measuring fungicide sensitivity

**Figure 3:**
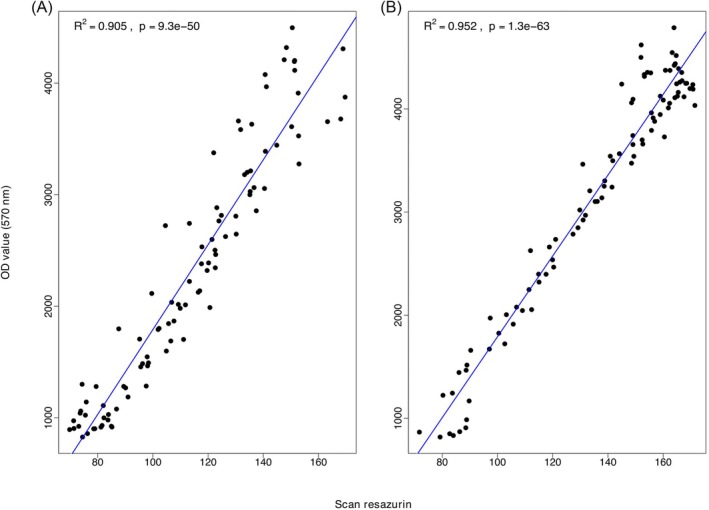
Correlation between biomass values (96 data points per panel) obtained in microtiter plate assays combining resazurin dye, a flatbed scanner, and image analysis compared with optical density at 570 nm on a plate reader. Eight serial dilutions of prothioconazole were distributed across the rows of 96 well microtiter plates and 11 different *Zymoseptoria tritici* isolates were distributed across the columns in each plate. Each plate contained one control column not inoculated with fungal spores. (A) Correlations using the concentration range of 0, 5, 10, 25, 50, 100, 150, 200 mg L^−1^. (B) Correlations using the concentration range of 0, 0.5, 5, 15, 30, 70, 120, 150 mg L^−1^.

Replications of the same isolate showed the same degree of red color intensity over different fungicide concentrations (Supporting Information, Fig. S2). EC_50_ values of biological replicates were compared to check the reproducibility of the method. Linear regression was calculated using a Bayesian model in SPSS.^37^ The resulting *R* ^2^ ranged from 0.98 to 0.99 (*P* < 0.05) for each tested AI. A direct comparison was made between the biomass values calculated using the new RZ method and the conventional OD method for 22 of the isolates. The correlation was high (*r* ^2^ = 0.90 and 0.95, *P* < 0.0001; Fig. 3).

Significant pairwise correlations (Pearson *P* < 0.001) were found between EC_50_ data sets for all AIs (Table 2), but the highest correlations were found among propiconazole, epoxiconazole and metconazole, suggesting that cross‐resistance may occur more readily among these DMIs. Cross‐resistance between different DMI AIs was already reported in *Z. tritici* ^2^ and *Alternaria alternata*.^40^ Cross‐resistance was also reported earlier between SDHI and azole fungicides in *Z. tritici*,^41^ in line with our results. The EC_50_ for each fungicide was calculated separately for the C1 and C3 collections of isolates, providing an average across replicates for 410 and 595 isolates, respectively (Supporting Information, Table S2). The mean (and range) EC_50_ values in mg L^−1^ were: 0.39 (0.01–6.53) for bixafen; 24.7 (0.01–127.60) for epoxiconazole; 17.99 (0.07–118.20) for metconazole; 26.79 (0.42–161.30) for propiconazole; 18.29 (0.01–99.88) for prothioconazole; and 4.34 (0.48–25.46) for spiroxamine (Table 3). The variance component analysis, using a linear mixed effects model, identified a significant broad‐sense heritability (*H* ^2^ = 0.53) associated with the EC_50_ values (Supporting Information, Table S3). A similar range of EC_50_ values was reported for bixafen by several researchers.^4^, ^43^ However, Birr *et al*.^41^ reported that the EC_50_ of propiconazole did not increase between 1999 and 2020 while also reporting a continuing decrease in sensitivity to prothioconazole, with a maximum EC_50_ value of <10 mg L^−1^. Torriani and coworkers^2^ reported EC_50_ values for prothioconazole ranging from 0.01 to 100 mg L^−1^, which is in line with our results. Similarly, Heick *et al*.,^42^ reported a range from 0.01 to 100 mg L^−1^ for prothioconazole in the Nordic–Baltic region. By contrast, the EC_50_ of epoxiconazole ranged from 0.01 to 10 mg L^−1^.^42^ However, in another report, one isolate had an EC_50_ value >100 mg L^−1^ for epoxiconazole.^19^ In summary, the EC_50_ values we recorded using RZ dye largely overlapped with the values reported by other laboratories.

**Table 2:** Correlation matrix between fitted EC_50_ data sets for six active ingredients. The upper diagonal shows the correlation coefficients and *P* values in parentheses

	EC_50_ Bixafen	EC_50_ Epoxiconazole	EC_50_ Metconazole	EC_50_ Propiconazole	EC_50_ Prothioconazole	EC_50_ Spiroxamine
EC_50_ Bixafen		0.55 (1.11 x 10^−71^)	0.54 (9.27 x 10^−69^)	0.70 (3.30 x 10^−61^)	0.15 (6.01 x 10^−6^)	0.47 (2.03 x 10^−50^)
EC_50_ Epoxiconazole			0.70 (1.46 x 10^−132^)	0.70 (5.83 x 10^−136^)	0.23 (3.00 x 10^−12^)	0.48 (2.19 x 10^−53^)
EC_50_ Metconazole				0.73 (7.33 x 10^−155^)	0.29 (5.73 x 10^−19^)	0.49 (2.69 x 10^−55^)
EC_50_ Propiconazole					0.26 (1.93 x 10^−15^)	0.52 (4.24 x 10^−65^)
EC_50_ Prothioconazole						0.16 (6.31 x 10^−7^)
EC_50_ Spiroxamine						

EC_50_, effective concentration of a fungicide that reduces growth by 50%.

**Table 3:** Minimum, maximum and mean EC_50_ values for six active ingredients for the C1 and C3 collections individually and combined

Active ingredient	Collection	Bixafen	Epoxiconazole	Metconazole	Propiconazole	Prothioconazole	Spiroxamine
Minimum	C1	0.01	0.19	0.07	0.42	0.23	0.48
	C3	0.01	0.01	0.11	0.51	0.01	0.58
Maximum	C1	6.53	127.60	118.20	161.30	99.88	25.46
	C3	2.00	127.20	111.90	151.70	89.59	21.04
Mean	C1	0.52	34.50	22.19	31.29	18.44	4.62
	C3	0.28	17.99	14.97	23.59	18.18	4.15
Mean	C1 and C3	0.39	24.70	17.99	26.79	18.29	4.34
SD	C1 and C3	0.46	26.78	18.27	26.35	12.72	2.94

EC_50_, effective concentration of a fungicide that reduces growth by 50%.

### 3.3 The effects of collection time on EC_50_

The EC_50_ values were significantly higher in the first collection (Table 3), with the same pattern found across most AIs. When using the EC_50_ for each replicate, the EC_50_ was significantly higher in the first collection for all AIs except spiroxamine (Supporting Information, Table S4). When using mean EC_50_ across replicates, the effect of collection time was significant for all but two AIs: spiroxamine and prothioconazole (Supporting Information, Table S5). Hence, we included collection time in our models to control for these differences.

### 3.4 The effects of H–F–P interactions on EC_50_

**Figure 4:**
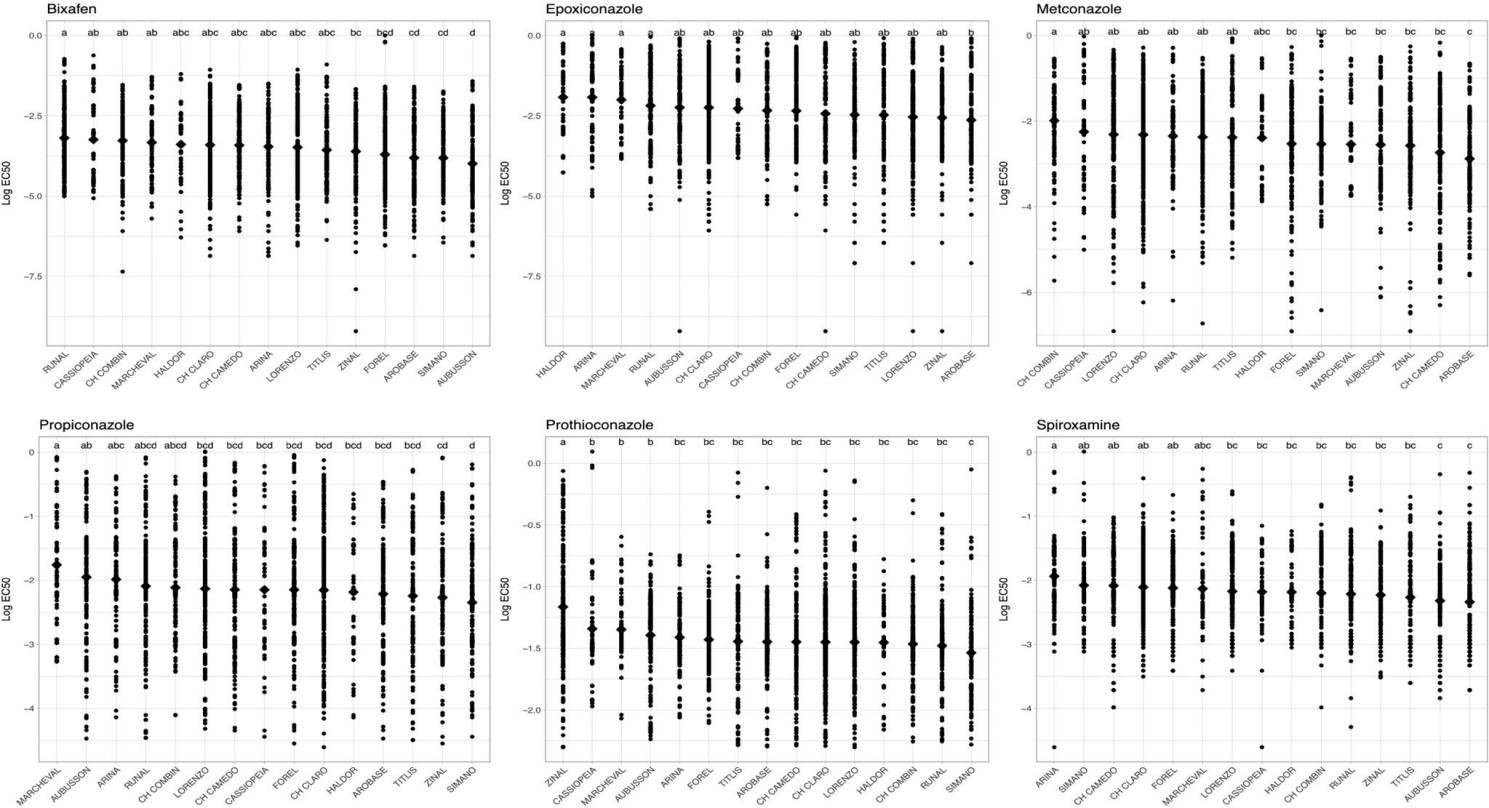
Log transformed and normalized EC_50_ values (points) for isolates sampled from different cultivars, sorted from highest mean EC_50_ (diamonds) to the lowest mean EC_50_. Letters atop each bar (i.e. cultivar) identify significantly different groups. Each panel includes data from the indicated fungicide. EC_50_, effective concentration of a fungicide that reduces growth by 50%.

*Z. tritici* isolations were made from 17 host genotypes that differed in their resistance to STB. We excluded the cultivars Garcia and Florett from this analysis because only 2 and 5 isolates, respectively, came from these cultivars, leaving 15 cultivars for comparisons. A significant H–F–P interaction was found when data with all AIs was combined: host, AI and their interaction explained a significant amount of the variation in the EC_50_ of each isolate. When considering each AI separately and each replicate as an observation, we found that EC_50_ varied with host cultivar for all AIs (Supporting Information, Table S4; Fig. 4). Individual cultivars hosted isolates with significantly higher EC_50_ values for an AI compared with other cultivars; for example, isolates from Zinal showed the highest EC_50_ for prothioconazole, whereas isolates from Cassiopeia showed higher EC_50_ values for both bixafen and metconazole (Fig. 4). If we use the mean across replicates, fewer significant host differences were observed; host did not explain variation in EC_50_ of propiconazole, and for epoxiconazole the effect was almost significant (0.056) (Supporting Information, Table S5 and Fig. S3).

Next, we tested for associations between the average EC_50_ for an AI on each cultivar with the average degree of STB resistance measured on that cultivar for three different measures of resistance: PLACL, ρlesions and ρleaf, using data reported previously.^9^ The STB resistance data sets were generated using the same leaves which were the source of the *Z. tritici* isolates described in this paper. We found negative correlations between the average EC_50_ values and the average degree of STB susceptibility measured for each cultivar for 14 of the 18 comparisons (Table 4), but only 3 of these were statistically significant. We note here that higher values for PLACL and pycnidia density reflect higher susceptibility to STB (Supporting Information, Table S2), whereas higher values for EC_50_ reflect higher resistance to the associated AI. Hence, a negative correlation between these factors indicates a positive relationship between STB resistance and resistance to the corresponding fungicide.

**Table 4:** Correlations between cultivar‐based EC_50_, i.e. mean EC_50_ value of isolates obtained from a certain cultivar, and Septoria tritici blotch resistance (PLACL, ρlesions, and ρleaf)

	PLACL	ρlesions	ρleaf	EC_50_ Bixafen	EC_50_ Epoxiconazole	EC_50_ Metconazole	EC_50_ Propiconazole	EC_50_ Prothioconazole	EC_50_ Spiroxamine
PLACL		0.25 (0.37)	**0.68 (0.01)**	−0.27 (0.34)	−0.06 (0.84)	**−0.61 (0.01)**	0.22 (0.43)	−0.17 (0.55)	−0.01 (0.99)
ρlesions			**0.70 (0.00)**	−0.41 (0.12)	−0.31 (0.26)	−0.33 (0.24)	0.19 (0.50)	0.21 (0.45)	**−0.56 (0.03)**
ρleaf				−0.44 (0.10)	−0.26 (0.36)	**−0.59 (0.02)**	0.15 (0.59)	−0.07 (0.79)	−0.36 (0.19)
EC_50_ Bixafen					0.45 (0.09)	**0.77 (0.00)**	0.47 (0.08)	0.13 (0.64)	0.21 (0.45)
EC_50_ Epoxiconazole						0.35 (0.20)	0.42 (0.12)	−0.34 (0.22)	0.43 (0.11)
EC_50_ Metconazole							0.23 (0.40)	0.04 (0.88)	0.05 (0.87)
EC_50_ Propiconazole								0.00 (0.99)	0.30 (0.28)
EC_50_ Prothioconazole									−0.20 (0.47)
EC_50_ Spiroxamine									

EC_50_, effective concentration of a fungicide that reduces growth by 50%; PLACL, percentage of leaf area covered by lesions; ρlesions, number of pycnidia found per cm^2^ of lesion; ρleaf, number of pycnidia found per cm^2^ of leaf. Correlation coefficients and *P* values (in parentheses) are in the upper diagonal, with significant values in bold. Tests were performed only for strictly independent variables.

## 4 DISCUSSION

*Z. tritici* is already known to adapt rapidly to its local environment by becoming virulent on genetically resistant cultivars^44^, ^45^ and resistant to applied fungicides.^46^ In this study, we developed a new method to measure EC_50_ values and used it to characterize the evolution of fungicide resistance during a single growing season in a single naturally infected field planted with a diverse collection of elite winter wheat cultivars. This field was treated three times during the growing season with five different AIs representing the SDHI, DMI, and morpholine modes of action. Despite the three fungicide treatments, the field populations maintained a high degree of variability for sensitivity to all five AIs over time. We found evidence for significant levels of resistance against all tested AIs. We also found strong evidence for significant host–fungicide–pathogen interactions.

### 4.1 RZ dye in microtiter plates provides a new method for measuring EC_50_ values in *Z. tritici*

Under stress, *Z. tritici* strains often increase melanin production^12^ and shift from blastospore growth to hyphal growth.^47^ Both processes can affect growth measurements based on the OD of solutions of fungal spores, so we developed a different method that measures overall metabolic activity as a proxy for fungal growth. Fungicide sensitivity in microtiter plates was based on the RZ color conversion from blue to red associated with total metabolic activity. This method provides quantitative EC_50_ measurements that are less likely to be affected by the type of growth (blastospores *versus* hyphal growth) or the production of melanin under stress. The method is semi‐automated and less prone to human error and has the advantage that the analyzed images can be archived for further analyses if questionable patterns emerge for particular strains, or if improved methods for image analysis are developed. We also note that flatbed scanners are typically much less expensive, more accessible, and easier to use than spectrometers and their associated microtiter plate readers. Although we compared the RZ and OD methods for only 22 of the tested strains, we found a high correlation between the indicated biomasses in each well, suggesting that the new method delivers results that largely align with the older method. A high correlation (*R* ^2^ = 0.99, *P* < 0.0001) was also found in earlier work that compared the RZ method with mycelial growth rates for ten strains of *M. fructicola* growing at a single diagnostic concentration of fenbuconazole.^28^ The EC_50_ of *Alternaria alternata* was also calculated using RZ (*R* ^2^ = 0.92; *P* < 0.0001).^23^ Other laboratories have validated the accuracy of the RZ method in comparison with the conventional OD method using various species, including 13 isolates of *Mycosphaerella fijiensis* and 14 isolates of *Pyricularia oryzae* that were tested against azoxystrobin, tebuconazole, and fluxapyroxad.^48^ The EC₅₀ values obtained by Silva *et al*.^48^ were in strong agreement with those previously reported using the OD method (*R* ^2^ = 0.99 *P* < 0.0001), offering additional support for the reliability of the RZ‐based approach.

### 4.2 A high diversity of fungicide sensitivities was maintained within a field population over time

The natural field populations of *Z. tritici* sampled in this experiment maintained very high diversity for fungicide sensitivity phenotypes, even after three cycles of selection due to fungicide applications. Despite the strong fungicidal selection, there was no evidence for increasing levels of fungicide resistance over time. The EC_50_ values ranged over two to four orders of magnitude across the >1000 isolates sampled from this naturally infected field. The range of EC_50_ values found in this single field during a single growing season is similar to the range in EC_50_ values found for the same fungicides at 55 field trial sites distributed across Europe over a 4‐year period.^46^ This agrees with the fine spatial scale over which phenotypic diversity is distributed within field populations of *Z. tritici*, as described in earlier publications.^10^, ^49^ This very high diversity is expected in natural populations not exposed to fungicide selection,^7^ but it was much higher than we expected to find in a field population exposed to three separate fungicide sprays composed of five AIs applied at full recommended dosages. All three applications were composed of fungicide mixtures, a widely recommended strategy to reduce directional selection for higher resistance.^50^ We consider it possible that use of fungicide mixtures contributed to the high phenotypic diversity found in this field population and may also contribute to the absence of higher resistance in the C3 sample compared to the C1 sample.

The density distribution of EC_50_ values followed a log‐normal distribution for all AIs (Supporting Information, Fig. S4) as commonly observed for many natural phenomena.^51^ A similar distribution of fungicide resistance was observed in field populations of the pathogens *Botrytis cinerea, Pyricularia graminis‐tritici*, and *Fusarium graminearum*.^20^, ^52^, ^53^ For these other fungi, large numbers of isolates were sampled from many different fields distributed across large geographical areas and often over several years. It was not reported in the other studies how intense was the fungicide selection pressure associated with each field collection. We consider it noteworthy that we found a similar distribution of fungicide sensitivities within a single field and during a single growing season in an environment that we expected would select strongly for fungicide resistance. This illustrates well that *Z. tritici* field populations can maintain a high evolutionary potential even after several cycles of strong selection. We consider it likely that the recombination resulting from sexual reproduction contributed to maintaining the high phenotypic diversity and high evolutionary potential in these treated populations.

The EC_50_ values showed a heritability of 0.53, consistent with an underlying genetic basis for these traits (Supporting Information, Table S3). We found a high correlation between the three replications of our experiment, indicating that the data were highly reproducible. Positive and high correlations between EC_50_ values for propiconazole, epoxiconazole, and metconazole provided evidence for cross‐resistance among these DMIs (Table 2). Similar cross‐resistance had already been reported between propiconazole and different DMIs in *Z. tritici* ^2^ and *Alternaria alternata*.^40^

In a previous analysis, Karisto *et al*.^9^ estimated, based on the recorded daily temperatures and daily records of precipitation, that two cycles of asexual reproduction occurred during the 43 days separating the C1 and C3 sampling dates. Fungicide mixtures were applied twice during this period, at 5 and 16 days after C1. Despite the evidence for a high heritability and the possibility for two cycles of selection, the mean EC_50_ values for all AIs were higher in C1 than in C3 (Table 3). This suggests that the fungicide applications occurring after C1 did not create a more‐resistant fungal population. This counterintuitive finding was unexpected, so we next describe two different processes that could function alone or together to explain the pattern.

#### 4.2.1 Process 1: Escape from selection

In this process, a significant fraction of the *Z. tritici* population was not exposed to a sufficiently high dose of an AI to kill the sensitive strains. The effective period of control for any fungicide ranges between 12 and 19 days, depending on the AI and temperature.^54^ *Z. tritici* is a necrotroph that continues to colonize the killed leaf tissue, as well as naturally senescent leaf tissue, and can persist on the lower senesced wheat leaves for many months. We hypothesize that fungicides applied to the top three or four green leaf layers of the canopy did not reach lethal concentrations in the senesced lower leaf layers. In this case, ascospores and pycnidiospores produced by fungicide‐sensitive isolates in the lower leaf layers would be able to initiate new infections on the upper leaf layers after the fungicide concentrations fell below lethal levels in the treated upper leaves.

#### 4.2.2 Process 2: High gene flow from untreated populations

In this process, a large number of airborne ascospores coming from nearby wheat fields that were not treated with fungicides introduced a high frequency of fungicide‐sensitive strains into the upper leaf layers of the experimental plots between C1 and C3, diluting the selected fungicide resistant strains. An earlier study reported that untreated wheat fields maintained *Z. tritici* populations with lower EC_50_ values than those isolated from fungicide‐treated fields.^16^ Previous studies showed that airborne ascospores coming from both outside a field and from lower leaves within a field can provide a significant source of inoculum on upper leaf layers during the later stages of an STB epidemic.^55^, ^56^

### 4.3 The H–F–P interaction

Wheat cultivars with different genetic backgrounds and expressing different degrees of STB resistance are known to directly impact the establishment of infection and the frequency of sexual and asexual reproduction.^57^, ^58^ In this experiment we found abundant evidence that different host cultivars produced populations of pathogen isolates expressing different degrees of fungicide sensitivity; i.e. there were significant H–F–P interactions (Fig. 4, Supporting Information, Fig. S3, Tables S4 and S5). The cultivar effect on AI resistance varied considerably and in each case a different subset of cultivars differed significantly from one another. Almost all cultivar‐isolate subgroups behaved differentially for AI resistance as indicated by EC_50_ values, suggesting that the cultivar genotype interacts with the fungicide environment to select the most adapted pathogen isolates. As examples, the isolates sampled from Zinal exhibited a significantly higher EC_50_ for prothioconazole, whereas isolates sampled from Runal were more resistant to bixafen and isolates from Arina were more resistant to spiroxamine (Fig. 4).

In general, positive relationships were found between the degree of resistance to STB measured on each cultivar and the degree of fungicide resistance in the *Z. tritici* isolates obtained from that same cultivar, although the correlations were significant only for metconazole and bixafen (Table 4). It is important to note here that the leaves used to obtain the measures of STB resistance are the same leaves that were used to obtain pathogen isolates, so the same pathogen populations were used to make the measurements of host STB resistance and pathogen fungicide resistance. Our findings suggest that more‐resistant hosts, on average, select for pathogen isolates that are more resistant to fungicides. This pattern was first described in populations of *Z. tritici* isolated from a resistant wheat cultivar and a susceptible wheat cultivar growing in the same Oregon field that was not treated with fungicides.^29^ In the same paper, a significant correlation was found between the virulence of 141 global *Z. tritici* isolates and their resistance to the DMI cyproconazole. The authors proposed that genes with pleiotropic effects could explain these correlations. Pathogen strains having the ability to detoxify or efflux the defense compounds (e.g. phytoalexins) produced by resistant hosts may also have the ability to detoxify or efflux synthetic antimicrobial compounds (e.g. fungicides), leading to a simultaneous increase in virulence and fungicide resistance. As an example, individual ABC transporters in *Botrytis cinerea* were shown to export both phytoalexins and fungicides.^59^ Yang^29^ also proposed that pathogens can produce defensive metabolites that can destroy or modify the structures and functions of both natural and synthetic antimicrobials. An example of such a defense metabolite is melanin, which has been shown to both increase virulence and reduce susceptibility to antimicrobial compounds for many pathogenic fungi.^60^ A significant correlation between the degree of melanization and fungicide sensitivity was found in a QTL mapping population of *Z. tritici*, with a QTL for melanization exactly overlapping with a QTL for sensitivity to propiconazole, suggesting that these traits are controlled by the same gene.^12^ Other analyses that included a global collection of 145 *Z. tritici* strains identified fitness trade‐offs between cultivar STB resistance and fungicide sensitivity, with significant negative genetic correlations found between resistance to propiconazole and both virulence and pathogen reproduction for 3 of the 12 tested wheat cultivars.^10^ A significant correlation was also found between melanization and fungicide sensitivity for these strains. This paper also reported specific SNPs that were associated with fungicide sensitivity, discovering genes related to known and candidate effectors as well as the *CYP51* gene involved in ergosterol synthesis. Although none of the earlier experiments were designed to test for H–F–P interactions, they provided evidence for a broad array of mechanisms that could contribute to putative H–F–P interactions. The new data set reported here came from an experiment that was designed to test for H–F–P interactions. Our results suggest that different hosts selectively favor pathogen isolates carrying resistance to different fungicides. This type of information may eventually prove useful for determining which fungicides should be applied to which cultivars to reduce the risk of emergence of fungicide resistance.

## 5 CONCLUSIONS

Our experiment showed that a high variability for fungicide sensitivity can be maintained in a pathogen population at the field scale even after three treatments with fungicide mixtures. More‐resistant wheat cultivars on average selected for pathogen strains with higher fungicide resistance. There was strong evidence for host–fungicide–pathogen interactions in which particular host genotypes selected for pathogen strains that were more resistant to particular AIs, suggesting that some host cultivars may accelerate the emergence of resistance to some fungicides.

## Supporting information


**Table S1.** Concentrations of active ingredients used in a second round of data collection for fungal isolates that needed different ranges of concentrations to calculate their EC50 values.

**Table S2.** Commercial names of winter wheat cultivars used in the field experiment combined with their STB resistance ranking (lower values indicate higher resistance), followed by the number of *Zymoseptoria tritici* isolates used in the current study. C1 sampling took place on 20 May and C3 sampling took place on 4 July. Overall ranking was based on ρleaf, the number of pycnidia found per cm^2^ of leaf. ρlesions is the number of pycnidia found per cm^2^ of lesion. PLACL is the percentage of leaf area covered by lesions.

**Table S3.** Variance component analysis for the effects of fungal isolate, host cultivar, and active ingredient on EC50 values. The broad‐sense heritability (*H* ^*2*^) was calculated based on the genetic *vs*. phenotypic variance.

**Table S4.** Results of modelling the effect of collection time, replicate and host cultivar on EC50 for each active ingredient. *P < 0.05, **P < 0.01, ***P < 0.001.

**Table S5.** Results of modelling the effect of collection time and host cultivar on mean EC50 across replicates for each active ingredient. *P < 0.05, **P < 0.01, ***P < 0.001.

**Figure S1.** Optimizing blastospore concentration involved a factorial experiment with two strains of *Z. tritici* (c1_25_2B2 and c1_290_9H1), each repeated twice. Least significant difference analysis based on ANOVA was conducted for both isolates. The black dots represent the mean of the red color scores (across the 8 blastospore concentrations) for the 12 different RZ concentrations that were tested for each blastospore concentration.

**Figure S2.** Examples of scanned microtiter plate wells used to calculate EC50 values for prothioconazole using resazurin dye. Each set of three columns represents one isolate of *Zymoseptoria tritici* with three replications (one column per replication). Rows 1 to 8 encompass serial dilutions of the active ingredient, ranging from zero fungicide (control) in the top row to the highest fungicide concentration in the bottom row. The circular areas indicated with yellow lines within the images denote the zones measured for red color intensity. Strips from different replicate plates were combined using Photoshop to display all three replications for each of the two isolates.

**Figure S3.** Log transformed and normalized EC50 values (grey points) for isolates sampled from different cultivars, sorted from highest mean EC50 (black diamond) to lowest. Distinct letters atop each bar (i.e., cultivar) signify statistically significant groups.

**Figure S4.** Density distribution of EC50 values for the tested active ingredients. The datasets include bixafen (A), epoxiconazole (B), metconazole (C), propiconazole (D), prothioconazole (E), and spiroxamine (F). The horizontal axes represent the range of EC50 values for the active ingredients in mg/l.

**File S1.** Script to generate QR code on Avery stickers (65 stickers: 38 X 21.2 mm).

**File S2.** Script for reading QR codes and generating the necessary folders for microtiter plate analyses.

**File S3.** Script for analyzing the digital photos of microtiter plates.

## Data Availability

The data that support the findings of this study are available on request from the corresponding author. The data are not publicly available due to privacy or ethical restrictions.
