# Supplementary figures and images for: A new method to measure EC50 reveals cultivar‐specific fungicide resistance and very high diversity within experimental field populations of Zymoseptoria tritici
Source: Pest Manag Sci. 2026 Jan 19;82(4):3613–24. doi: 10.1002/ps.70483 (PMC12976203; doi:10.1002/ps.70483)

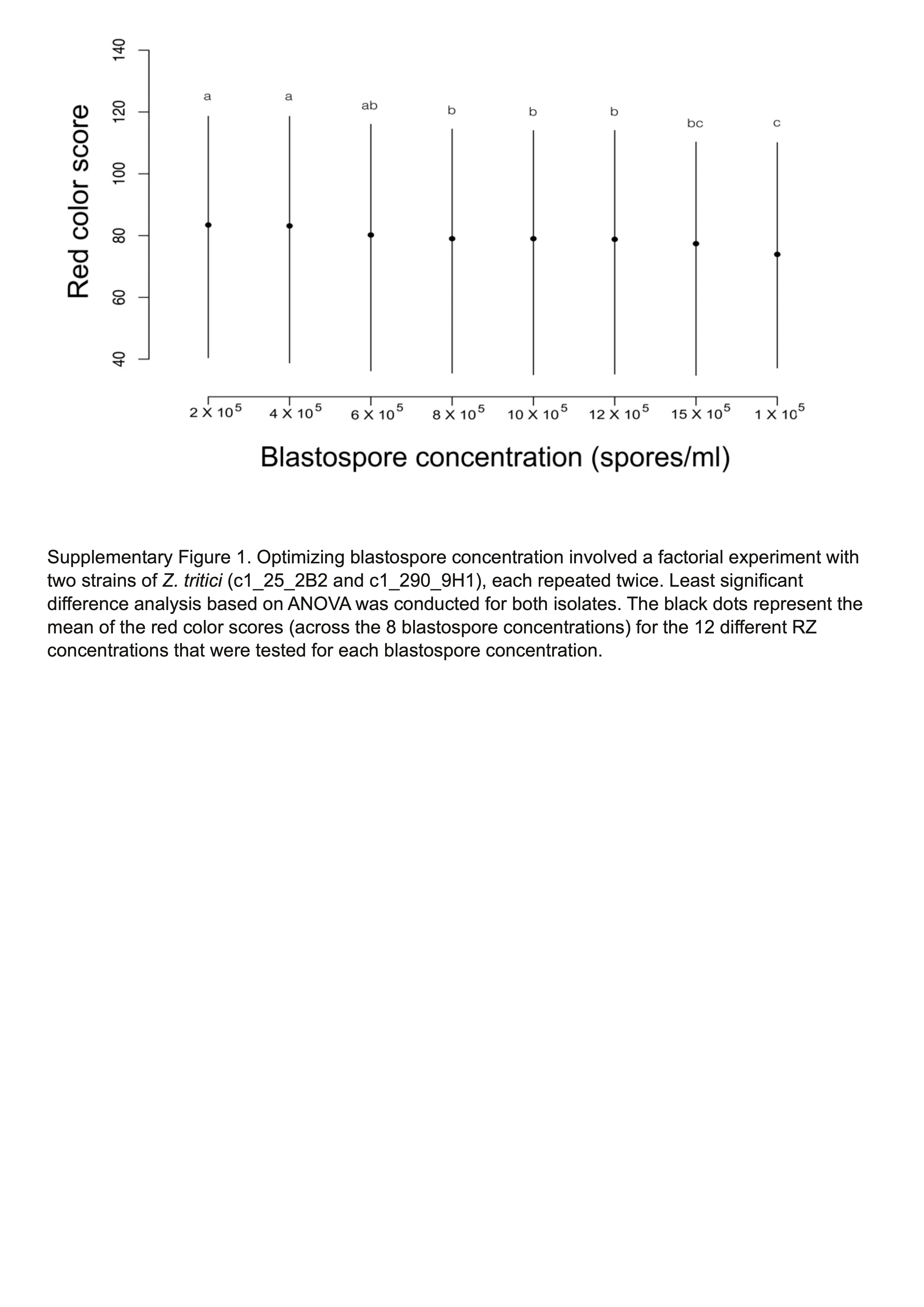

Supplement: Supplementary file 6 — Figure S1. Optimizing blastospore concentration involved a factorial experiment with two strains of Z. tritici (c1_25_2B2 and c1_290_9H1), each repeated twice. Least significant difference analysis based on ANOVA was conducted for both isolates. The black dots represent the mean of the red color scores (across the 8 blastospore concentrations) for the 12 different RZ concentrations that were tested for each blastospore concentration. [file PS-82-3613-s001.jpg]

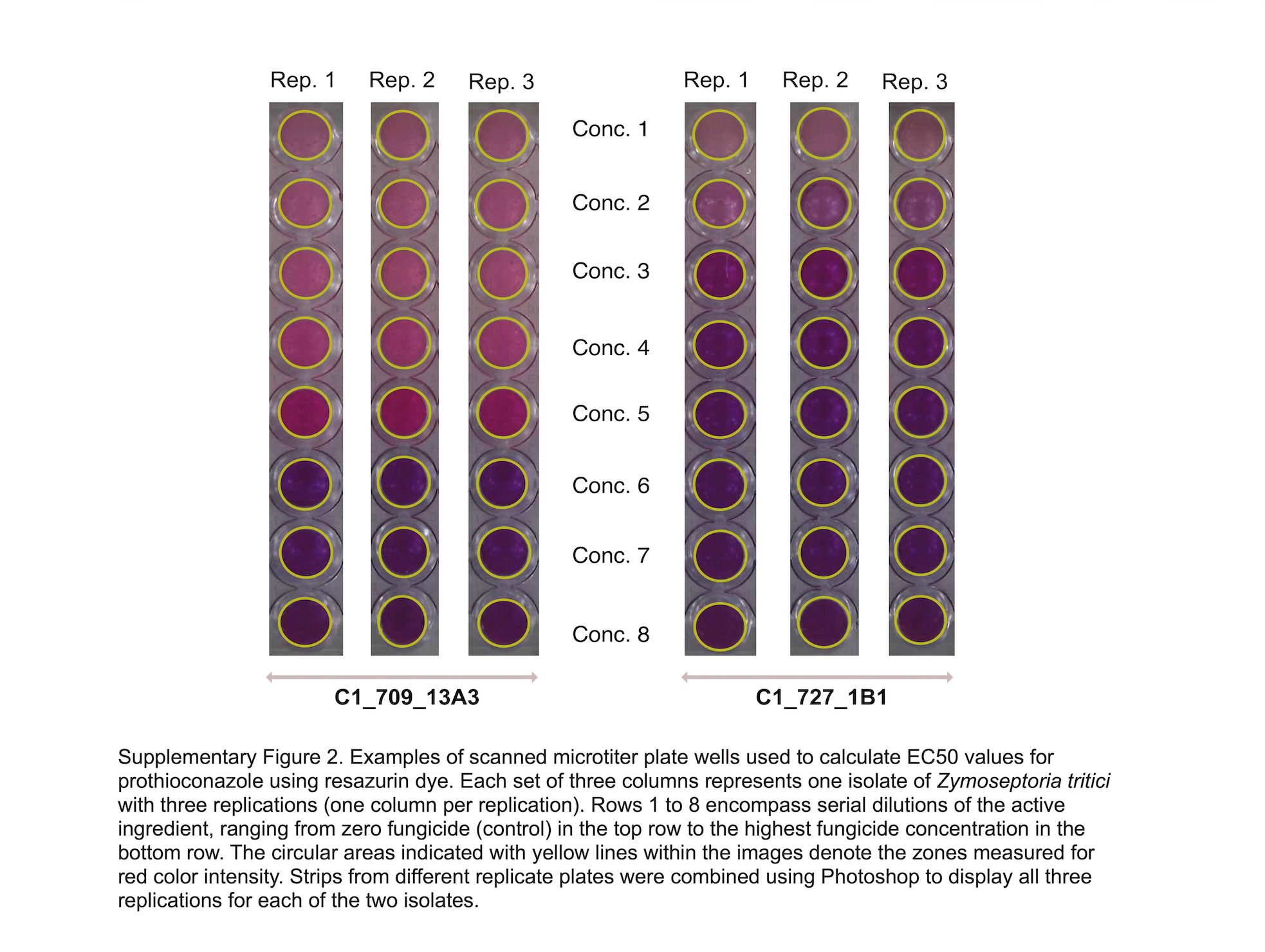

Supplement: Supplementary file 7 — Figure S2. Examples of scanned microtiter plate wells used to calculate EC50 values for prothioconazole using resazurin dye. Each set of three columns represents one isolate of Zymoseptoria tritici with three replications (one column per replication). Rows 1 to 8 encompass serial dilutions of the active ingredient, ranging from zero fungicide (control) in the top row to the highest fungicide concentration in the bottom row. The circular areas indicated with yellow lines within the images denote the zones measured for red color intensity. Strips from different replicate plates were combined using Photoshop to display all three replications for each of the two isolates. [file PS-82-3613-s005.jpg]

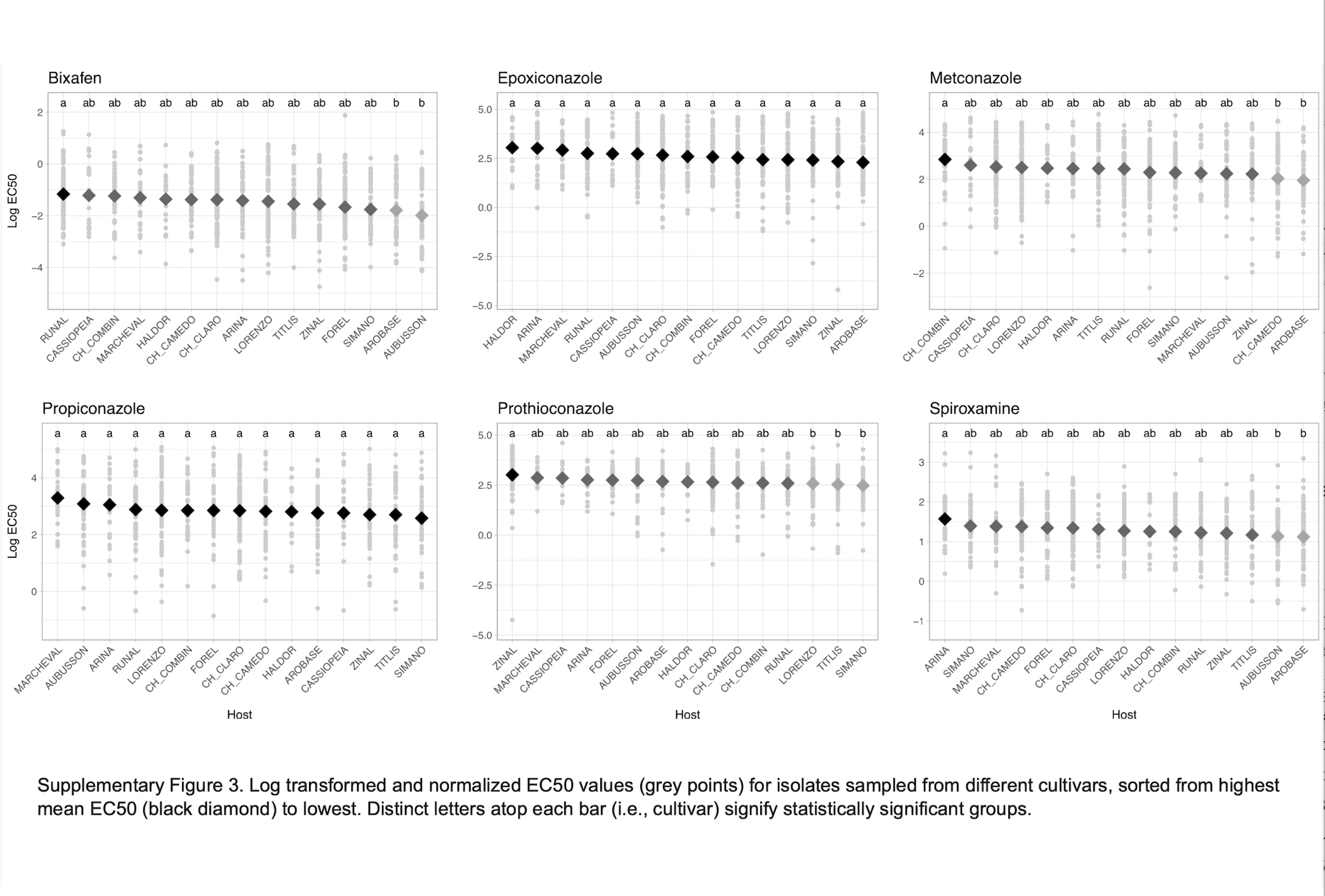

Supplement: Supplementary file 8 — Figure S3. Log transformed and normalized EC50 values (grey points) for isolates sampled from different cultivars, sorted from highest mean EC50 (black diamond) to lowest. Distinct letters atop each bar (i.e., cultivar) signify statistically significant groups. [file PS-82-3613-s007.jpg]

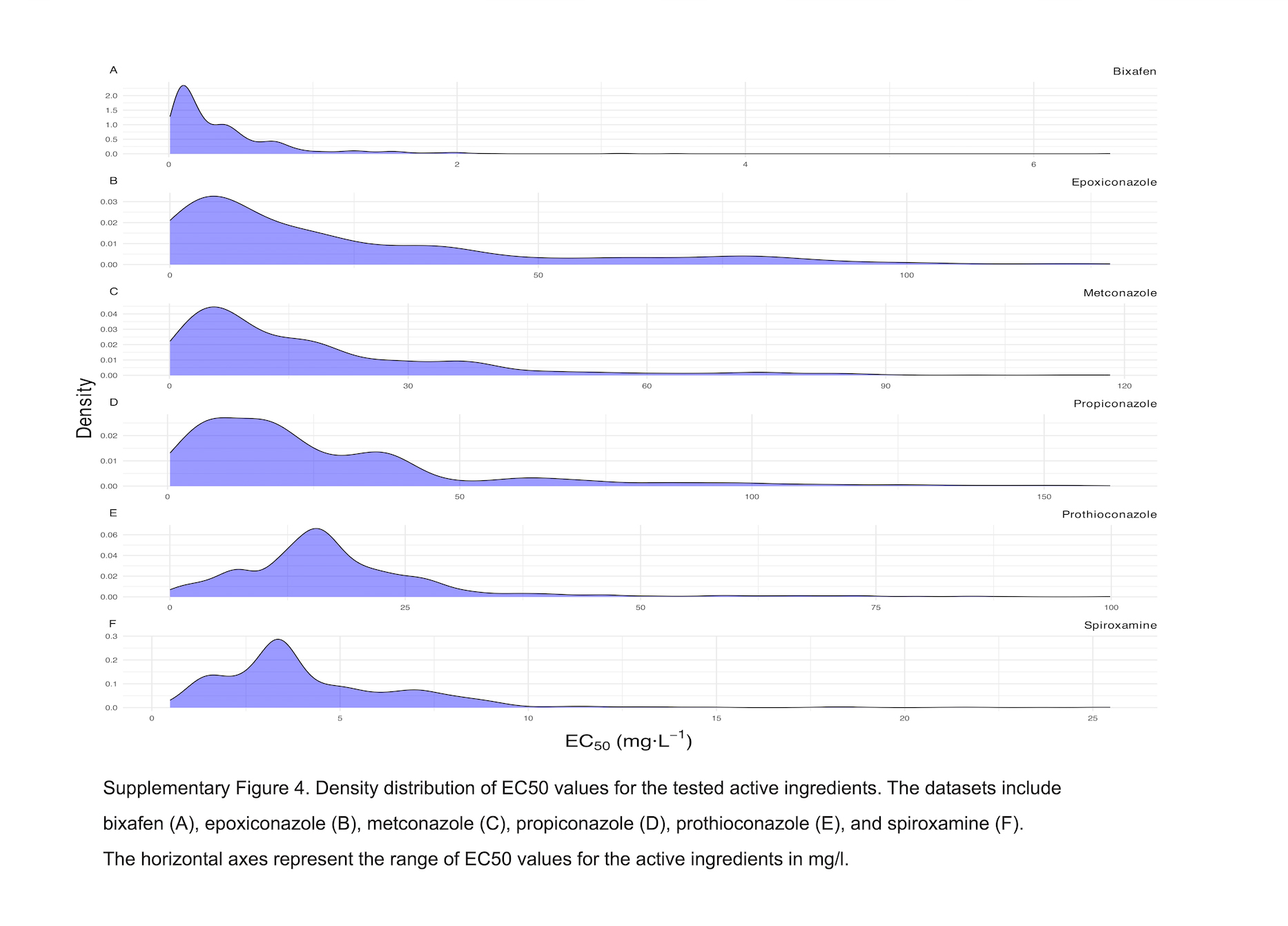

Supplement: Supplementary file 9 — Figure S4. Density distribution of EC50 values for the tested active ingredients. The datasets include bixafen (A), epoxiconazole (B), metconazole (C), propiconazole (D), prothioconazole (E), and spiroxamine (F). The horizontal axes represent the range of EC50 values for the active ingredients in mg/l. [file PS-82-3613-s012.jpg]
